# No Correlation Exists between Disease Activity and the Expression of Killer-Cell Immunoglobulin-Like Receptors in Patients with Rheumatoid Arthritis

**DOI:** 10.1155/2007/65179

**Published:** 2006-12-27

**Authors:** Toshiaki Kogure, Takeshi Tatsumi, Atsushi Niizawa, Hiroshi Fujinaga, Tomoyuki Ito, Yutaka Shimada, Katsutoshi Terasawa

**Affiliations:** ^1^Department of Integrated Japanese Oriental Medicine, School of Medicine, Gunma University, 3-39-22 Showa-Machi, Maebashi 371-8511, Japan; ^2^Department of Japanese Oriental Medicine, Kanebo Memorial Hospital, Misakicho 1-9-1 Hyogo-Ku, Kobe, Hyogo 652-0855, Japan; ^3^Department of Japanese Oriental Medicine, Toyama Prefectural Central Hospital, 2-2-78 Nishinagae, Toyama 930-8550, Japan; ^4^Department of Internal Medicine (Rheumatology), Nagaoka Red-Cross Hospital, 297-1 Terajima-Machi, Nagaoka, Niigata 940-2085, Japan; ^5^Department of Japanese Oriental Medicine, Faculty of Medicine, University of Toyama, 2630 Sugitani, Toyama-Shi, Toyama 930-0194, Japan; ^6^Department of Japanese Oriental Medicine, Graduate School of Medicine, Chiba University, Chiba 260-8670, Japan

## Abstract

*Objective*. The genes for killer-cell immunoglobulin-like receptors (KIRs) have been cloned and their functions and expression in patients with rheumatoid arthritis (RA) have been partially clarified. However, the correlation between their expression and disease activity has not been analyzed in patients with RA. Thus, we measured KIR expression on lymphocytes in patients with RA, and assessed the correlation between KIR expression and disease activity. *Patients and Methods*. In the cross-sectional study, 15 patients (9 females and 6 males) who fulfilled the diagnostic criteria for RA were assessed. In the longitudinal study, patients who were followed-up for 3 months were assessed. CD158a/b expression on peripheral blood mononuclear cells (PBMC) of RA patients was analyzed using flow cytometry. *Results*. No significant correlation between KIR expression and CRP, ESR, or IgM-RF was observed. There was no remarkable change in the expression of KIRs between the baseline and after 3 months. Additionally, in the 5 patients whose expression of KIRs particularly changed, the time-related changes in the expression of KIRs were independent from those of inflammation parameters and IgM-RF. *Conclusion*. There was no correlation between KIR expression and disease activity; therefore, the clinical use of KIR expression should be limited, while unnatural KIR expression may be involved in the pathogenesis of RA, but not a recruitment of chronic inflammation to induce joint damage.

## 1. INTRODUCTION

The molecular cloning of novel NK receptors (NKRs) was reported [1–3], and it was subsequently shown that the receptors transmit negative signals [4, 5]. In human, NKRs can be divided into 3 classes: immunoglobulin (Ig)-like receptors, including killer cell Ig-like receptors (KIRs) and leukocyte Ig-like receptors (LIRs), lectine-like receptors (CD94/NKG), and natural cytotoxicity receptors (NCRs), including NKp30, NKp44, and NKp46 [6].

KIRs are expressed on many NK cells and on subpopulations of T-cells [7], and have been implicated in autoimmune conditions such as rheumatoid arthritis (RA) [8, 9] and scleroderma [10], where associations have been found with 3-domain KIRs (KIR2DL1, 2, and 3) known to recognize HLA-C alleles [11, 12]. We have also demonstrated that the expression of KIR2DL1 on CD8+ T cells was reduced in patients with RA compared to healthy subjects, and that the upregulation of KIR2DL1, 2, and 3 by treatment with IL-2 was weaker in RA patients than in healthy subjects [9]. Low KIR expression on CD8+ T cells in RA might be associated with the mechanism of self-attacking characteristics of autoimmune diseases. Also, the low response of KIRs to IL-2 suggests that patients with RA might be highly susceptible to *Mycobacterium tuberculosis* (TB). Based on these findings, it is considered that the status of KIR expression may be involved in the pathogenesis of RA as well as susceptibility to microbes such as TB [13].

RA is a chronic destructive joint disease and a heterogeneous syndrome. Therefore, the disturbance of KIR expression in RA raises an interesting question: does KIR expression on peripheral lymphocytes in patients with RA change with disease activity or not? However, changes in KIR expression on lymphocytes have not yet been analyzed in RA patients.

Here, we measured KIR expression on lymphocytes in patients with RA, and assessed the correlation between KIR expression and disease activity and changes in KIR expression during the disease course.

## 2. PATIENTS AND METHODS

### 2.1. Patients

Fifteen patients (9 females and 6 males) with flares of RA as defined by the revised criteria of the American College of Rheumatology [14] were included in this study. The characteristics of these patients were as follows: mean ± SD age of 62.0 ± 9.4 years (range: 42–72), mean ± SD disease duration of 8.1 ± 6.2 years (range: 2–24), mean ± SD ESR (erythrocyte sedimentation rate) 48 ± 35 mm/h (range: 28–86), mean ± SD CH50 (serum complement titer/50% hemolytic unit of complement) 36.9 ± 7.0 U/mL (range: 28–52), anatomical stage [15] 2.2 ± 1.4, and functional class 2.1 ± 0.5. All were receiving nonsteroidal anti-inflammatory drugs (NSAIDs). Two were also taking Bucillamine, 7 Salazosulfapyridine, and 4 Prednisolone (PSL, 2.5–7.5 mg/day). Eight patients were receiving Methotrexate. No patient was receiving Infliximab.

### 2.2. Reagents and cells

Fluorescein-isothiocyanate (FITC)-conjugated antihuman CD16, phycoerythrin (PE)-conjugated antihuman CD158a (EB6), and PE-conjugated antihuman CD158b (GL183) were purchased from Immunotech, Marseille, France. Antibody EB6 reacts with KIR2DL1 and KIR2DS1, and GL183 reacts with KIR2DL2, KIR2DL3, and KIR2DS2 [11, 12].

Peripheral blood mononuclear cells (PBMCs) obtained from patients with RA were separated from heparinized blood by Lymphoprep (Nyegaard, Oslo, Norway) gradient centrifugation [16]. Each PBMC was incubated in a culture dish in a humidified 5% CO_2_/95% air atmosphere at 37°C for 60 minutes. After incubation, nonadherent cells were collected. These cell suspensions were washed twice in phosphate-buffered saline (PBS).

### 2.3. Cell phenotype

Surface phenotyping was carried out using a two-color immunofluorescence staining technique, with isotype-specific mouse antihuman antibody conjugated with either FITC or PE [17]. Each sample of stained cells was suspended in 0.5 mL of PBS and analyzed by flow cytometry. Lymphocyte subsets were identified by gating analysis, and fluorescence profiles were obtained for 10000 cells from each sample. Negative controls for each experiment were performed with FITC- and PE-labeled mouse immunoglobulin-G (Ig-G).

### 2.4. Statistical analysis

Data are expressed as mean (SD) values. All data were collected in a computer database and analyzed using the StatView-J 4.02 program (Abacus Concept, Berkeley, Calif, USA). The Mann-Whitney *u* test was performed for each set of the surface antigen. For all statistical tests, differences were regarded as significant if *P* < .05.

## 3. RESULTS

The correlation between the counts of KIR-expressing cells and disease activity in RA.

The correlation between CD158a/b-expressing cells and CRP, ESR, and IgM-rheumatoid factor (IgM-RF) is summarized in Table 1. There was no correlation between the counts of CD158a-expressing cells and disease activity, although there was a tendency towards a correlation between CD158a-expressing cells and ESR. Also, the counts of CD158b-expressing cells did not correlate with classical inflammatory parameters (CRP: C-reactive protein, and ESR) and IgM-RF.

The changes of CD158a- and CD158b-expressing cells during the followup of 3 months.

**Figure 1:**
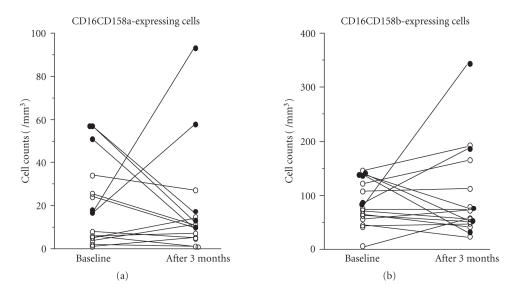
Changes in the counts of CD158a- and CD158b-expressing cells during the followup of 3 months: (a) CD158a cells and (b) CD158b cells. The majority of RA patients did not show changes in the numbers of CD158a- and CD158b-expressing cells. Five patients (•) underwent an expansion or reduction in the populations of both CD158a- and CD158b-expressing cells. However, time-related changes in the expression of CD158a/b were independent from those of inflammation parameters and IgM-RF in the 5 cases.

Figure 1 shows the changes in the populations of cells expressing CD158a (Figure 1(a)) and CD158b (Figure 1(b)) during the followup of 3 months. Ten of the RA patients did not show changes in the expression of CD158a and CD158b. Two patients showed an expansion of the populations of both CD158a- and CD158b-expressing cells. In contrast, 3 patients showed decrease in the populations of both CD158a- and CD158b-expressing cells. However, time-related changes in the expression of CD158a/b were independent from those of inflammation parameters and IgM-RF in the 5 cases (Figure 1). Additionally, there were no differences in clinical features among the three groups: (i) patients with no changes in KIR expression, (ii) patients with a decrease in KIR expression, and (iii) patients with an increase in KIR expression (data not shown).

## 4. DISCUSSION

The biological functions of KIRs have been thoroughly investigated. In brief, NK cell function is generally regulated by a balance between signals transmitted by inhibitory and stimulatory receptors. Ligand binding to inhibitory receptors recruits phosphatases that dephosphorylate downstream activation proteins, thereby terminating the activating-signaling pathway. Thus, activation of NK cell function occurs when there is a net excess of stimulatory over inhibitory signals [18].

Recent studies have demonstrated findings supporting the important functions of NK cells in regulating autoimmunity [19]. We have also demonstrated that the population of CD8+CD158a+ cells was reduced in patients with RA compared to healthy subjects [9]. Similarly, Nakiri et al have reported that among patients with RA, NKB1+CD8+ T cells decreased significantly in comparison to controls [20]. It is considered that the low population of KIR-expressing cells in T cells might be associated with the mechanism of self-attacking characteristics of autoimmune diseases, and that an unnatural expression of KIRs may contribute to the pathogenesis of RA. However, it has not been demonstrated whether KIR expression correlates with disease activity in RA.

As far as we know, this is the first report to assess the correlation between KIR expression and disease activity. In this study, we did not find a significant relationship between CD158a/b expression on peripheral lymphocytes, classical inflammatory parameters (CRP and ESR), and IgM-RF. These findings suggest that unnatural KIR expression contributes to one of the triggers of RA pathogenesis, but not a recruitment of chronic inflammation to induce joint damage. Other investigators have revealed that the KIR2DS2 gene was significantly enriched among patients with vasculitis in RA [8].

Furthermore, we have observed the changes in the population of CD158a/b-expressing cells. In this followup, time-related changes in the population of KIR-expressing cells were independent from those of inflammation parameters and IgM-RF. Additionally, the patients were divided into 3 groups according to the pattern of changes in the population of KIR-expressing cells. However, there was no difference in the clinical features among the 3 groups: (i) patients with no changes in KIR expression, (ii) patients with a decrease in KIR expression, and (iii) patients with an increase in KIR expression. We also did not find any correlation in the disease activity between these 3 groups. These findings may also suggest that unnatural KIR expression contributes to one of the triggers of RA pathogenesis, but not a recruitment of chronic inflammation to induce joint damage. To support this hypothesis, further analysis of KIR expression on the cells of several lineages is required.

Finally, there was no correlation between KIR expression and disease activity; therefore, the clinical use of KIR expression should be limited, while unnatural KIR expression may be involved in the pathogenesis of RA, but not a recruitment of chronic inflammation to induce joint damage.

## Figures and Tables

**Table 1:** The correlation between the counts of KIRs-expressing cells and disease activity in RA.

CD158a	Probability	Spearman's rank correlation coefficient

CRP*	0.386	0.241
ESR**	0.052	0.517
IgM-RF^#^	0.411	0.239

CD158b	Probability	Spearman's rank correlation coefficient

CRP	0.991	0.003
ESR	0.651	0.127
IgM-RF	0.939	0.023

*CRP: C-reactive protein.

**ESR: erythrocyte sedimentation rate.

^#^IgM-RF: IgM-rheumatoid factor.
